# The non-syndromic familial thoracic aortic aneurysms and dissections maps to 15q21 locus

**DOI:** 10.1186/1471-2350-11-143

**Published:** 2010-10-11

**Authors:** Ali R Keramati, Anita Sadeghpour, Maryam M Farahani, Gurangad Chandok, Arya Mani

**Affiliations:** 1 Department of Internal Medicine, Yale University School of Medicine, New Haven, CT, 06511 USA; 2 Department of Echocardiography, Rajaie Cardiovascular Medical and Research Center, Iran University of Medical Sciences, Tehran, 19668 Iran; 3 Penn State University Hershey Medical Center, Hershey, PA, 17036 USA

## Abstract

**Background:**

Thoracic aortic aneurysms and dissections (TAAD) is a critical condition that often goes undiagnosed with fatal consequences. While majority of the cases are sporadic, more than 20% are inherited as a single gene disorder. The most common familial TAA is Marfan syndrome (MFS), which is primarily caused by mutations in *fibrillin-1 *(*FBN1*) gene. Patients with *FBN1 *mutations are at higher risk for dissection compared to other patients with similar size aneurysms.

**Methods:**

Fifteen family members were genotyped using Affymetrix-10K genechips. A genome-wide association study was carried out using an autosomal dominant model of inheritance with incomplete penetrance. Mutation screening of all exons and exon-intron boundaries of *FBN1 *gene which reside near the peak Lod score was carried out by direct sequencing.

**Results:**

The index case presented with agonizing substernal pain and was found to have TAAD by transthoracic echocardiogram. The family history was significant for 3 first degree relatives with TAA. Nine additional family members were diagnosed with TAA by echocardiography examinations. The affected individuals had no syndromic features. A genome-wide analysis of linkage mapped the disease gene to a single locus on chromosome 15q21 with a peak Lod score of 3.6 at *fibrillin-1 *(*FBN1*) gene locus (odds ratio > 4000:1 in favour of linkage), strongly suggesting that *FBN1 *is the causative gene. No mutation was identified within the exons and exon-intron boundaries of *FBN1 *gene that segregated with the disease. Haplotype analysis identified additional mutation carriers who had previously unknown status due to borderline dilation of the ascending aorta.

**Conclusions:**

A familial non-syndromic TAAD is strongly associated with the *FBN1 *gene locus and has a malignant disease course often seen in MFS patients. This finding indicates the importance of obtaining detailed family history and echocardiographic screening of extended relatives of patients with non-syndromic TAAD to improve the outcome. In addition, association of non-syndromic TAAD with the Marfan disease gene locus poses the question whether secondary prevention strategies employed for Marfan syndrome patients should be applied to all patients with familial TAAD.

## Background

Thoracic aortic aneurysms and dissections (TAAD) is a lethal condition with a rising incidence [1,2]. Although this condition often occurs sporadically, about one out of every 5 cases is familial. This includes syndromic forms of the disease, including Marfan syndrome (MFS) and the less commonly seen Loeys-Dietz syndrome as well as the non-syndromic thoracic aortic aneurysm. The mode of inheritance in most cases is consistent with autosomal dominant with incomplete penetrance[3]. It is a heterogeneous disorder for which more than 4 loci have been mapped [4-7].

Marfan syndrome is a connective tissue disorder that affects multiple organs including cardiovascular and skeletal systems [7]. It is often inherited as an autosomal dominant disorder caused by *FBN1 *gene mutation [8-12]. Its most lethal complication is aortic aneurysm, a malignant disorder which results in early onset dissection and death [13]. Whether *FBN1 *mutation can cause non-syndromic familial aneurysm and more importantly if the disease course is equally malignant is not known. Absence of syndromic features in *FBN1 *mutation carriers may not have implications for disease screening. This screening may include obtaining detailed family and an echocardiographic examination. In addition, the family history and the disease course in the family members with advanced disease and complications may impact the future planning for at risk family members. With the advent of cost effective high throughput sequencing techniques, mutation screening of patients with thoracic aortic aneurysms for known genes such as *FBN1 *may become part of the routine clinical workup.

In the current study, we identified a large three-generation family with multiple members affected by early onset TAAD in absence of syndromic features. A genome-wide analysis of linkage was carried out to map the disease gene.

## Methods

### Family Characterization

The research protocol was approved by the institutional review boards of all participating institutions. All study participants provided written informed consent for clinical and genetic studies. Detailed family history was obtained and extensive physical examination by a clinical geneticist was carried out. Family members were examined for musculoskeletal, pulmonary, and dermatological features of MFS based on Ghent nosology. Ophthalmologic and echocardiographic examinations were carried out by an ophthalmologist and a cardiologist, respectively. For echocardiographic evaluation of the aneurysms, diameters of the aorta at the sinus of valsalva, the supra-aortic ridge, and the aortic root were measured from cross-sectional echocardiography images in the parasternal long-axis. Individuals were identified as affected if they had an aneurysm (diameter ≥3.6 cm at the aortic root or supra-aortic ridge level) or dissection of the ascending thoracic aorta. MRI examinations of the lumbosacral region were carried out on limited number of patients with thoracic aneurysms. Blood samples were collected from family members. Genomic DNA was isolated from 15 family members.

### Genotyping and Analysis of Linkage

Thirteen family members were called as affected (2 were deceased), 2 as unaffected and 2 as unknown. Affymetrix 10K DNA genechip arrays were used to genotype > 10,000 SNPs across the genome in all 15 living family members.

Allele frequencies for each SNP were mean allele frequencies of 20 unrelated unaffected Iranian (ethnically matched) and penetrance was set at 90%. Multipoint analysis of linkage with Genehunter was used for the genome scan analysis.

### Sequence variation detection and functional analysis

All 66 exons and exon-intron boundaries of *FBN1 *were amplified by PCR and were evaluated using direct dye-termination sequencing. Sequences were evaluated using the sequencer software and were interpreted independently by two lab members. Complementary strands were sequenced to confirm potential substitutions. Skin biopsies were obtained as part of the clinical workup by the local dermatologists and were evaluated by the local diagnostic laboratory using immunocytochemistry.

## Results

### Clinical Evaluations

**Figure 1:**
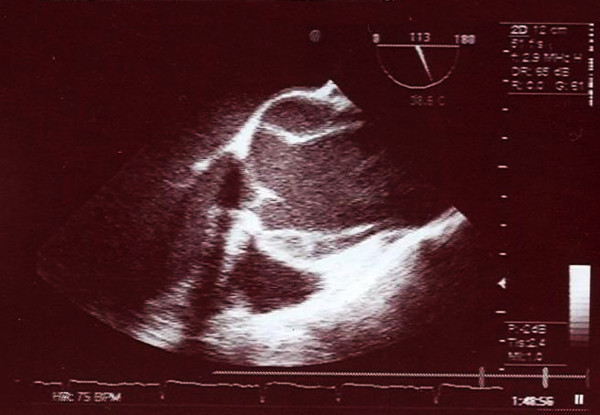
**Ascending aortic dissection**. Transthoracic echocardiogram in index case depicting dissection in proximal ascending aorta.

**Figure 2:**
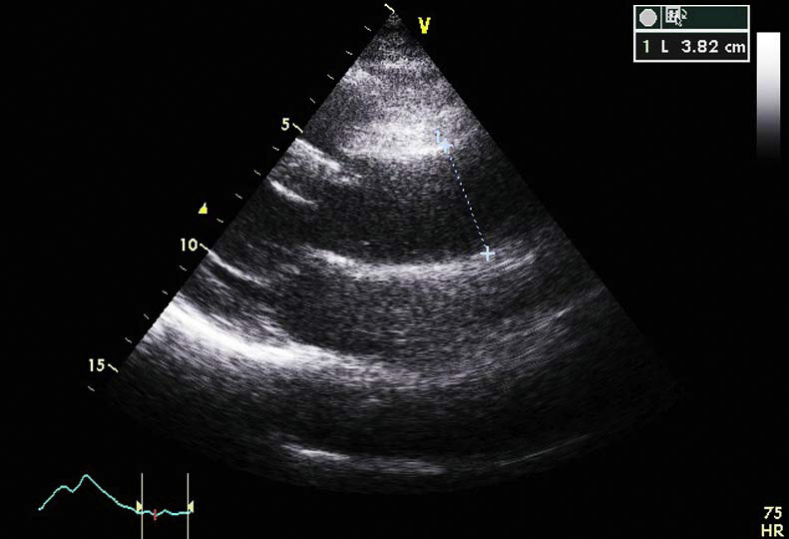
**Echocardiography image-aneurysm**. A left parasternal view of the ascending aorta in echocardiography examination of an affected family member with an aneurysm radius of 3.8.

We studied a 3-generation Iranian family. The Index case (individual 6) was a 55 years old woman who presented with acute substernal chest pain. The echocardiographic examination revealed normal left ventricular wall motion but dilated aorta with a proximal ascending aorta diameter of 5.5 cm with evidence for dissection (Figure 1). Patient was only 170 cm tall, and did not exhibit disproportionately long extremities compared with the trunk (dolichostenomelia); no evidence for arachnodactyly was noted. Wrist (Walker) and thumb (Steinberg) signs were absent and no other musculoskeletal, pulmonary, ophthalmological or dermatological abnormalities of the Ghent nosology were identified. Immediate surgical repair was planned, but the patient deceased on the way to the operating room. Her older sister had presented at the age of 51 years with dissection of a 5 cm ascending aortic aneurysm and the younger sister had been diagnosed with proximal ascending aortic dilation involving the aortic root with moderate aortic regurgitation and both had undergone Bentall procedures. Interestingly, the son of the index case presented later with TAAD and had to undergo Bentall procedure. Ten additional immediate family members were classified as affected and 2 as unaffected by echocardiographic examinations (aortic root or ascending aortic diameter ≥ 3.6 cm, Figure 2). Two other individuals (14 and 17) had aortic diameters (3.2 and 3.1 cm) that were considered relatively large for their age but did not exceed the cut off for diagnosis and were assigned the unknown status. No syndromic feature suggestive of MFS was detected in any of the family members (Additional File 1).

### Mapping of the Locus

**Figure 3:**
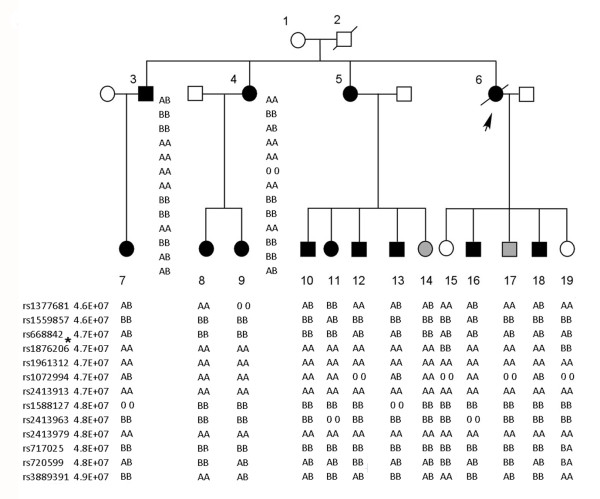
**TAAD pedigree**. Relationships of members of kindred with TAAD are shown. The index case is indicated by the arrow. Numbered individuals correspond to those in Additional File 1. Individuals with TAA are indicated by black symbols; individuals without TAA are shown as unfilled symbols; and individuals who have unknown status are shown as half-gray symbols. Circles represent females; squares represent males. Symbols with a slash through them indicate deceased subjects. Genotypes of informative SNP markers are shown in their chromosomal order below the symbol for each individual and their distances from 15pter are indicated in megabases.

**Figure 4:**
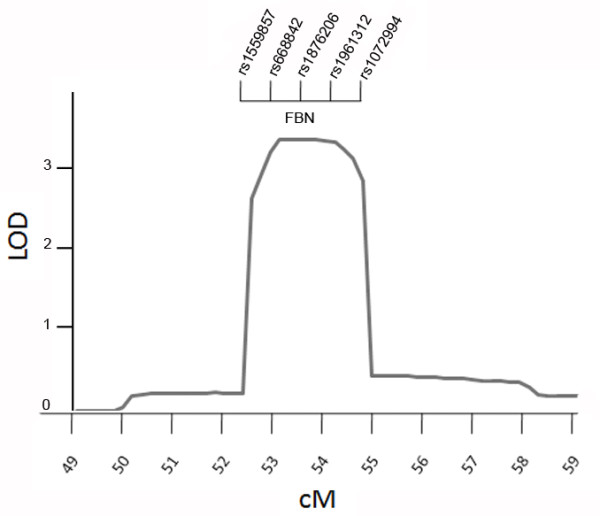
**Lod Score**. Multipoint lod scores for linkage of TAAD to 15q21. SNPs tightly linked to the location of the maximum lod score are indicated and the location of *FBN1 *is shown. The lod score peak occurs at zero recombination with marker rs1876206. *y*-Axis, LOD score; *x*-axis, position of the markers on chromosome 15q, based on their relative chromosomal distance. Position of the markers is given in centiMorgans (cM).

A genome-wide analysis of linkage in 15 family members (11 affecteds, 2 unaffecteds and 2 unknowns) was carried out (Figure 3). The model was specified as autosomal dominant with 90% penetrance, 1% phenocopy rate, and disease allele frequency of 0.001. It was run on Genehunter 2.0 software. The disease gene for TAAD in this kindred was mapped to a single interval with a significant lod score that peaked at the *FBN1*gene locus (Lod = 3.6, θ = 0). No other interval had lod score > 1(Figure 4).

Construction of the 15q haplotype with SNPS revealed recombinations immediately prior to rs668842 and after rs1072994, refining the linked interval to less than 500 kb. Haplotype analysis indicated the segregation of the disease haplotype in two family members whose ascending aorta were assessed as borderline dilated, thus identifying two individuals with unknown status as mutation carriers.

### Mutation analysis and FBN1 expression

No disease segregating mutation was identified in exons or exon-intron boundaries of *FBN1 *genes. No other intronic mutation was identified which could potentially affect the splicing. A total of 5 novel and evolutionarily highly conserved intronic mutations were identified which did not segregate with the disease. Haplotype analysis of the affected individuals within this kindred showed that all the identified intronic mutations reside on unlinked/non-disease chromosomes and did not refine the linked interval.

The results from skin biopsies of 3 family members with TAAD were reported as suggestive for Marfan syndrome based on abnormal expression of FBN1 in fibroblasts. No images were provided to us by the laboratory.

## Discussion

Thoracic aortic aneurysms and dissections in patients with MFS has very poor outcome. Identification of at risk individuals prior to an event is therefore of great importance. In the current study, genome-wide analysis in a family with non-syndromic TAAD has mapped the disease gene to MFS disease gene locus. Although mutation carriers in this family did not have syndromic features of the Marfan syndrome, the aortic disease in many of the affected family members had a malignant course. This finding suggests that autosomal dominant non-syndromic TAAD may be associated with *FBN1 *gene mutation and implicates the necessity of echocardiographic screening of the relatives of patients with familial TAAD.

Acute aortic aneurysm is an uncommon but potentially catastrophic illness with an incidence of approximately 2.9/100,000/yr [14]. Predisposing factors are either acquired such as trauma, caused by complex traits like, hypertension, and bicuspid aortic valve or inherited such as in Marfan syndrome. Among these, patients with Marfan syndrome are especially at high risk for aortic dissection of the ascending aorta at a relatively young age [13]. Patients with familial aneurysms also tend to be younger and have higher rates of dissection compared to those with sporadic aneurysms [15], signifying the importance of early screening of at risk family members. An analysis of a large database of patients treated at Yale-New Haven Hospital [16] has shown that patients with familial autosomal dominant thoracic aortic aneurysms present with complications at much younger ages (mean 57 years compared to sporadic cases (mean age 64 years).

Although non-syndromic familial TAAD is a hereditary disorder, genetic causes of it remain vastly unknown. An investigation of the families of 158 patients referred for surgical repair of thoracic aortic aneurysms or dissections found that first-degree relatives of probands had a higher risk (risk ratio [RR] 1.8 for fathers and sisters, RR 10.9 for brothers) of thoracic aortic aneurysms or sudden death compared with control subjects [1].

At least four different loci have been thus far mapped for familial non-syndromic thoracic aortic aneurysms. Mutations in transforming growth factor-beta receptor type II have been associated with some cases of familial thoracic aortic aneurysms [5,6]. Mutations in myosin heavy chain (*MYH11*), a smooth muscle cell-specific contractile protein, have been identified in familial TAAD associated with patent ductus arteriosus (PDA) linked to 16p12.2-12.13[17]. However, some families have mapped to neither of these loci suggesting additional loci. We provide a strong evidence for linkage of non-syndromic familial thoracic aneurysms and dissections to *FBN1 *locus. Although a unique phenotype of aortic dissections involving both the ascending and descending aorta along has been mapped to chromosome 15q in a single kindred [18], we report here for the first time linkage of a large family with isolated TAAD to MFS gene locus. All affected individuals and none among the unaffecteds carry the disease haplotype. Association between *FBN1 *mutation and familial non-syndromic aortic aneurysm has been previously reported [19]. In the reported kindred, *FBN1 *mutation segregated in most but not all family members and there were mutation carriers who were unaffected. Moreover, some affected individuals had one or another musculoskeletal finding of Marfan syndrome. *FBN1 *mutation has been also reported in sporadic cases of non-syndromic thoracic aneurysm [20]. MFS is a pleiotropic disease and many of its phenotypes may be absent in one individual and be present in a close relative. Since the extended families of patients with TAAD were not examined for the musculoskeletal phenotypes of Marfan syndrome, diagnosis of MFS cannot be definitively excluded. Moreover, the proof of causality was based on a single mutation and not genetic linkage analysis.

In this study, we did not identify any disease segregating mutation in the *FBN1 *gene in the affected individuals. Although there may be an unidentified disease causing mutation within the linked interval unrelated to *FBN1 *gene, the small size of the linked interval and the significant lod score that peaks at *FBN1 *gene locus rather indicate that disease in this kindred is caused an unidentified variation within this gene. It is a general consensus that absence of *FBN1 *mutation does not exclude diagnosis because of high rates of false negative results, likely due to presence of intronic mutations. In addition, identification of nonsynonymous mutations in absence of significant linkage to the disease locus or clinical criteria is not considered diagnostic for MFS. There is increasing controversy about significance of many of the identified *FBN1 *mutations. *FBN1 *mutations can be found in many other conditions that may or may not overlap with Marfan syndrome such as skin and skeletal findings (Shprintzen-Goldberg syndrome)[21], syndrome of mitral valve prolapse, myopia, borderline aortic enlargement, skin and skeletal findings, Marfanoid habitus and isolated ectopia lentis. In contrast analysis of linkage remains one of the most reliable molecular methods for diagnosis of Marfan syndrome[22]. Most importantly, the lod-1 interval in this kindred contains only two other genes (*SLC12A1, DYUT*) which because of their tissue distributions and functions cannot be causative gene for TAAD.

## Conclusions

We demonstrate in this study the largest reported kindred with non-syndromic TAAD that unambiguously links to 15q21, the locus for *FBN1 *gene. We conclude that in genetic study of non-syndromic TAAD, *FBN1 *gene should be considered as one of the candidate genes. Moreover, the malignant course of the disease in this kindred underscores the importance of echocardiographic screening in extended relatives of patients with familial non-syndromic TAAD. Finally, it raises an important question to whether secondary prevention strategies employed for Marfan syndrome patients which include angiotensin converting enzyme inhibitors should be also examined in patients with early onset familial TAAD.

## Competing interests

The authors declare that they have no competing interests.

## Authors' contributions

ARK and GC did the genotyping and sequencing work and helped with manuscript preparation.

AS and MMF identified the kindred, conducted the clinical examination and phenotyping, obtained IRB approval and collected DNA.

AM designed and supervised the study and wrote the manuscript.

All authors read and approved the final manuscript.

## Pre-publication history

The pre-publication history for this paper can be accessed here:

http://www.biomedcentral.com/1471-2350/11/143/prepub

## Supplementary Material

Additional file 1 **Clinical features of the family members according to the Ghent Nosology**. 17 family members of kindred were examined for musculoskeletal, ocular, pulmonary, cardiovascular, ophthalmologic and dermatological features of Marfan syndrome based of Ghent Nosology. Click here for file

## References

[B1] Biddinger A Rocklin M Coselli J Milewicz D Familial thoracic aortic dilatations and dissections: a case control study J Vasc Surg 1997 25 3 506 511 10.1016/S0741-5214(97)70261-1 9081132

[B2] Olsson C Thelin S Ståhle E Ekbom A Granath F Thoracic aortic aneurysm and dissection: increasing prevalence and improved outcomes reported in a nationwide population-based study of more than 14,000 cases from 1987 to 2002 Circulation 2006 114 24 2611 2618 10.1161/CIRCULATIONAHA.106.630400 17145990

[B3] Milewicz D Chen H Park E Petty E Zaghi H Shashidhar G Willing M Patel V Reduced penetrance and variable expressivity of familial thoracic aortic aneurysms/dissections Am J Cardiol 1998 82 4 474 479 10.1016/S0002-9149(98)00364-6 9723636

[B4] Biggin A Holman K Brett M Bennetts B Adès L Detection of thirty novel FBN1 mutations in patients with Marfan syndrome or a related fibrillinopathy Hum Mutat 2004 23 1 99 10.1002/humu.9207 14695540

[B5] Loeys B Schwarze U Holm T Callewaert B Thomas G Pannu H De Backer J Oswald G Symoens S Manouvrier S Aneurysm syndromes caused by mutations in the TGF-beta receptor N Engl J Med 2006 355 8 788 798 10.1056/NEJMoa055695 16928994

[B6] Pannu H Fadulu V Chang J Lafont A Hasham S Sparks E Giampietro P Zaleski C Estrera A Safi H Mutations in transforming growth factor-beta receptor type II cause familial thoracic aortic aneurysms and dissections Circulation 2005 112 4 513 520 10.1161/CIRCULATIONAHA.105.537340 16027248

[B7] Murdoch J Walker B Halpern B Kuzma J McKusick V Life expectancy and causes of death in the Marfan syndrome N Engl J Med 1972 286 15 804 808 10.1056/NEJM197204132861502 5011789

[B8] Kainulainen K Pulkkinen L Savolainen A Kaitila I Peltonen L Location on chromosome 15 of the gene defect causing Marfan syndrome N Engl J Med 1990 323 14 935 939 10.1056/NEJM199010043231402 2402262

[B9] Dietz H Cutting G Pyeritz R Maslen C Sakai L Corson G Puffenberger E Hamosh A Nanthakumar E Curristin S Marfan syndrome caused by a recurrent de novo missense mutation in the fibrillin gene Nature 1991 352 6333 337 339 10.1038/352337a0 1852208

[B10] Tsipouras P Sarfarazi M Devi A Weiffenbach B Boxer M Marfan syndrome is closely linked to a marker on chromosome 15q1.5----q2.1 Proc Natl Acad Sci USA 1991 88 10 4486 4488 10.1073/pnas.88.10.4486 2034688 PMC51685

[B11] Lee B Godfrey M Vitale E Hori H Mattei M Sarfarazi M Tsipouras P Ramirez F Hollister D Linkage of Marfan syndrome and a phenotypically related disorder to two different fibrillin genes Nature 1991 352 6333 330 334 10.1038/352330a0 1852206

[B12] Dietz H Pyeritz R Hall B Cadle R Hamosh A Schwartz J Meyers D Francomano C The Marfan syndrome locus: confirmation of assignment to chromosome 15 and identification of tightly linked markers at 15q15-q21.3 Genomics 1991 9 2 355 361 10.1016/0888-7543(91)90264-F 2004786

[B13] Faivre L Collod-Beroud G Loeys B Child A Binquet C Gautier E Callewaert B Arbustini E Mayer K Arslan-Kirchner M Effect of mutation type and location on clinical outcome in 1,013 probands with Marfan syndrome or related phenotypes and FBN1 mutations: an international study Am J Hum Genet 2007 81 3 454 466 10.1086/520125 17701892 PMC1950837

[B14] Mészáros I Mórocz J Szlávi J Schmidt J Tornóci L Nagy L Szép L Epidemiology and clinicopathology of aortic dissection Chest 2000 117 5 1271 1278 10.1378/chest.117.5.1271 10807810

[B15] Hagan P Nienaber C Isselbacher E Bruckman D Karavite D Russman P Evangelista A Fattori R Suzuki T Oh J The International Registry of Acute Aortic Dissection (IRAD): new insights into an old disease JAMA 2000 283 7 897 903 10.1001/jama.283.7.897 10685714

[B16] Coady M Davies R Roberts M Goldstein L Rogalski M Rizzo J Hammond G Kopf G Elefteriades J Familial patterns of thoracic aortic aneurysms Arch Surg 1999 134 4 361 367 10.1001/archsurg.134.4.361 10199307

[B17] Zhu L Vranckx R Khau Van Kien P Lalande A Boisset N Mathieu F Wegman M Glancy L Gasc J Brunotte F Mutations in myosin heavy chain 11 cause a syndrome associating thoracic aortic aneurysm/aortic dissection and patent ductus arteriosus Nat Genet 2006 38 3 343 349 10.1038/ng1721 16444274

[B18] Tran-Fadulu V Chen J Lemuth D Neichoy B Yuan J Gomes N Sparks E Kramer L Guo D Pannu H Familial thoracic aortic aneurysms and dissections: three families with early-onset ascending and descending aortic dissections in women Am J Med Genet A 2006 140 11 1196 1202 16646045 10.1002/ajmg.a.31236

[B19] Francke U Berg M Tynan K Brenn T Liu W Aoyama T Gasner C Miller D Furthmayr H A Gly1127Ser mutation in an EGF-like domain of the fibrillin-1 gene is a risk factor for ascending aortic aneurysm and dissection Am J Hum Genet 1995 56 6 1287 1296 7762551 PMC1801106

[B20] Milewicz D Michael K Fisher N Coselli J Markello T Biddinger A Fibrillin-1 (FBN1) mutations in patients with thoracic aortic aneurysms Circulation 1996 94 11 2708 2711 8941093 10.1161/01.cir.94.11.2708

[B21] Sood S Eldadah Z Krause W McIntosh I Dietz H Mutation in fibrillin-1 and the Marfanoid-craniosynostosis (Shprintzen-Goldberg) syndrome Nat Genet 1996 12 2 209 211 10.1038/ng0296-209 8563763

[B22] Dietz H Molecular etiology, pathogenesis and diagnosis of the Marfan syndrome Prog Pediatr Cardiol 1996 5 159 166 10.1016/1058-9813(96)00161-0

