# Supplementary material for: The non-syndromic familial thoracic aortic aneurysms and dissections maps to 15q21 locus
Source: BMC Med Genet. 2010 Oct 11;11:143. doi: 10.1186/1471-2350-11-143 (PMC2958900; doi:10.1186/1471-2350-11-143)
Supplement: Additional file 1 — Clinical features of the family members according to the Ghent Nosology. 17 family members of kindred were examined for musculoskeletal, ocular, pulmonary, cardiovascular, ophthalmologic and dermatological features of Marfan syndrome based of Ghent Nosology. [file 1471-2350-11-143-S1.PDF]

Additional File-1 Clinical features of the family members according to the Ghent Nosology. A (N) sign donates absence, a (Y) sign donates presence and a (N/A) sign indicates that the status is unknown

Abbreviations; A= Age (Onset), H= Height (m), W= Weight (Kg), PC= Pectus carinatum, PE= Pectus excavatum, SH= Span to height >1.05, WT= Wrist and thumb signs, SC= Scoliosis >20°, SL=spondylolisthesis, EE= Elbow extension <170°, PP= Pes planus, PA= Protrusio acetabulae, OSF = Other skeletal finding including joint hypermobility, high arched palate, dental crowding and characteristic facial appearance, EL= Ectopia lentis, OOF= Other ocular findings including Flat Cornea, Increased axial length of the globe and hypoplasmic iris or ciliary muscle causing decreased miosis, DAA= Dilatation of ascending aorta involving at least sinuses of Valsalva (Cm), Dis= Dissection of ascending aorta, OCF=Other Cardiovascular Findings including Mitral Valve Prolase, dilatation of the main pulmonary artery, calcification of mitral annulus below the age of 40 years, calcification of mitral valve and dilatation or dissection of the descending thoracic or abdominal aorta below age of 50, PF= Pulmonary Findings including spontaneous pneumothorax and apical bleb, Skin Findings= steriae atrophicae and recurrent or incisional hernia, DE= Lumbosacral dural ectasia by CT or MRI, Fx= Family/genetic history, FD= First degree relative with Marfan's syndrome

ID No	A	H	W	PC	PE	SH	WT	SC	SL	EE	PP	PA	OSF	EL	OOF	DAA	Dis	OCA	PF	SF	DE	Fx	FD
3	57	1.73	78	N	N	N	N	N	N	N	N	N	N	N	N	3.9	N	N	N	N	N/A	N	N
4	54	1.69	73	N	N	N	N	N	N	N	N	N	N	N	N	5.6	N	N	N	N	N/A	N	N
5	51	1.65	67	N	N	N	N	N	N	N	N	N	N	N	N	5	Y	N	N	N	N/A	N	N
6	55	1.7	82	N	N	N	N	N	N	N	N	N	N	N	N	5.5	Y	N	N	N	N/A	N	N
7	32	1.67	76	N	N	N	N	N	N	N	N	N	N	N	N	3.7	N	N	N	N	N/A	N	N
8	31	1.67	70	N	N	N	N	N	N	N	N	N	N	N	N	4.2	N	N	N	N	N/A	N	N
9	34	1.65	70	N	N	N	N	N	N	N	N	N	N	N	N	4.2	N	N	N	N	N/A	N	N
10	30	1.59	65	N	N	N	N	N	N	N	N	N	N	N	N	4.2	N	N	N	N	N/A	N	N
11	29	1.72	78	N	N	N	N	N	N	N	N	N	N	N	N	3.8	N	N	N	N	N/A	N	N
12	32	1.7	72	N	N	N	N	N	N	N	N	N	N	N	N	4.6	N	N	N	N	N	N	N
13	33	1.72	72	N	N	N	N	N	N	N	N	N	N	N	N	4.3	N	N	N	N	N	N	N
14	27	1.67	71	N	N	N	N	N	N	N	N	N	N	N	N	3.2	N	N	N	N	N/A	N	N
15	34	1.73	72	N	N	N	N	N	N	N	N	N	N	N	N	2.6	N	N	N	N	N/A	N	N
16	47	1.65	69	N	N	N	N	N	N	N	N	N	N	N	N	N/A	Y	N	N	N	N/A	N	N
17	26	1.66	69	N	N	N	N	N	N	N	N	N	N	N	N	3.1	N	N	N	N	N/A	N	N
18	42	1.68	72	N	N	N	N	N	N	N	N	N	N	N	N	5.5	N	N	N	N	N	N	N
19	33	1.5	62	N	N	N	N	N	N	N	N	N	N	N	N	2.7	N	N	N	N	N/A	N	N
